# Quality of life and mood disorders

**DOI:** 10.1192/j.eurpsy.2021.517

**Published:** 2021-08-13

**Authors:** N. Smaoui, I. Lajmi, A. Guermazi, S. Omri, R. Feki, M. Maalej Bouali, N. Charfi, J. Ben Thabet, L. Zouari, M. Maalej

**Affiliations:** Psychiatry C Department, Hedi chaker University hospital, sfax, Tunisia

**Keywords:** quality of life, mood disorders

## Abstract

**Introduction:**

Many researches addressing quality of life (QOL) has been demonstrated its impairment during acute episodes of bipolar disorder (BD) and major depressive disorder (MDD).

**Objectives:**

To compare QOL between patients with remitted MDD and remitted BD and healthy controls (HC).

**Methods:**

A comparative and analytical study, conducted over 3 months in the outpatient psychiatric department of Hedi Chaker University Hospital in Sfax (Tunisia) among 30 patients with remitted BD, 30 patients with remitted MDD and 34 HC. QOL was assessed with the «36 item Short-Form Health Survey» (SF-36).

**Results:**

Compared with HC, the MDD and the BD groups had significantly lower scores for the total of the SF-36 and its sub-domains (table 1). Physical scores were lower in patients with MDD, compared with patients with BD (table 1). Table 1: Comparison of SF-36 sub-domain scores between MDD, BD patients, and HC.

**Table:** 

Sub-domains of the SF36	MDD	BD	HC	P
Mean physical score - Physical functioning - limitation due to physical health - Pain - General health	45.5 67 42.5 60 48.5	59,28 69,00 44,17 67,13 56,83	77,86 84,26 71,03 83,50 72,05	0.000 0.003 0.005 0.001 0.000
Mean psychic score - limitation due to emotional problems - Social functioning - Energy/fatigue - Emotional well-being	47.25 41 55.8 40 52	48,19 48.89 43.48 46.5 53.86	68,66 76.97 75.52 56.02 66.12	0,000 0.000 0.000 0.002 0.007
Mean global score	50.88	53,73	73,78	0,000

**Conclusions:**

QOL of patients with mood disorders such as MDD and BD suffered damage even in euthymic periods.

